# Virus Detection: A Review of the Current and Emerging Molecular and Immunological Methods

**DOI:** 10.3389/fmolb.2021.637559

**Published:** 2021-04-20

**Authors:** A. Cassedy, A. Parle-McDermott, R. O’Kennedy

**Affiliations:** ^1^ School of Biotechnology, Dublin City University, Dublin, Ireland; ^2^ Hamad Bin Khalifa University, Doha, Qatar; ^3^ Qatar Foundation, Doha, Qatar

**Keywords:** immunoassay, isothermal amplification, next generation sequencing, virus, nucleic-acid detection, sampling

## Abstract

Viruses are ubiquitous in the environment. While many impart no deleterious effects on their hosts, several are major pathogens. This risk of pathogenicity, alongside the fact that many viruses can rapidly mutate highlights the need for suitable, rapid diagnostic measures. This review provides a critical analysis of widely used methods and examines their advantages and limitations. Currently, nucleic-acid detection and immunoassay methods are among the most popular means for quickly identifying viral infection directly from source. Nucleic acid-based detection generally offers high sensitivity, but can be time-consuming, costly, and require trained staff. The use of isothermal-based amplification systems for detection could aid in the reduction of results turnaround and equipment-associated costs, making them appealing for point-of-use applications, or when high volume/fast turnaround testing is required. Alternatively, immunoassays offer robustness and reduced costs. Furthermore, some immunoassay formats, such as those using lateral-flow technology, can generate results very rapidly. However, immunoassays typically cannot achieve comparable sensitivity to nucleic acid-based detection methods. Alongside these methods, the application of next-generation sequencing can provide highly specific results. In addition, the ability to sequence large numbers of viral genomes would provide researchers with enhanced information and assist in tracing infections.

## Introduction

Viruses are ubiquitous entities which rely on host organisms to replicate. They exist in all habitats and are capable of infecting a wide range of life forms, from bacteria to plants and animals. Structurally, at a minimum, viruses consist of two core components. These are a nucleic acid genome, comprised of either double- or single-stranded DNA or RNA, and a capsid. The capsid is a highly symmetrical structure, formed by multiple copies of a small number of proteins and encoded by the viral genome (Domingo, 2015). The capsid packages the viral nucleic acid, protecting it. However, the capsid is also known to be multifunctional, with roles in cell-entry, genome uncoating or intracellular trafficking (Mateu, 2013; Freire et al., 2015). In addition to the capsid protein, some viruses will have an added layer of protection known as an envelope. This is generally comprised of a lipid or glycoprotein coating. Lipid envelopes are derived from the cell membrane of the infected host, whereas glycoprotein envelopes are typically coded for by the viral genome. The viral genome also encodes proteins which facilitate replication and assembly of the virus particles. An assembled, infectious, virus particle is termed a virion (Banerjee and Mukhopadhyay, 2016). While structurally, most viruses present similar characteristics, such as the symmetric capsid proteins, genetically, they are highly diverse.

This genetic diversity gives rise to a lack of understanding or an unawareness of many of the viruses present in our environment. While viruses dominate most habitats, the virome is far from complete. In conjunction with this, the interaction between host and virus remains unclear in many cases (Paez-Espino et al., 2016). While it is known that infection by some viruses imparts deleterious effects on their host, in other cases, the relationship between the two may be mutually beneficial (Pradeu, 2016; Jagdale and Joshi, 2018). It is feasible that many of these virus-host interactions, where the host remains unharmed, may persist unidentified, as infection may not present clearly recognisable symptoms. Linked to these unknown host-virus interactions is limited knowledge of the various host ranges of viruses. The host range is the number of species known to be used by a pathogen, however, defining the hosts which a virus may, or may not, infect is not easily feasible (McLeish et al., 2019). These unknown host ranges often play a role in emergence. In conjunction with this, other drivers of viral emergence are climate change, increased global transport, centralized agriculture and a high density of crops, animals and humans. These factors create an environment where the spread of viruses to hosts, which normally would not fall within the host range, is more easily facilitated. The flexibility and diversity of the viral genome itself is also a major factor driving diversification of viral host-ranges and environmental tolerances. Some viruses have the capacity to mutate at extremely rapid rates. For example, RNA viruses can have a mutation rate up to a million times higher than their hosts and can incorporate mutated nucleotides at a rate of 10^–6^–10^–4^ substitutions per nucleotide per cell infection (s/n/c) (Sanjuán and Domingo-Calap, 2016; Duffy, 2018). DNA viruses typically mutate at a slower rate than RNA viruses, with an estimated s/n/c of 10^–8^–10^–6^ (Sanjuán and Domingo-Calap, 2016). This ability of viruses to rapidly mutate and transfer between hosts makes the development of dynamic methods to detect current and emergent viral strains extremely pertinent.

Viral diagnostics are generally organised into two primary categories i.e., indirect and direct detection. This review aims to critically assess the currently employed methods for viral detection and examine their suitability, with due note to scenarios where assays may need to be rapidly developed, such as during epidemics or pandemics.

## Indirect Detection

Indirect detection methods involve the propagation of virus particles via their introduction to a suitable host cell line. This is termed virus isolation. Virus isolation remains a relevant and important method, particularly as it offers a means to propagate existing and emergent viruses for further analysis and characterisation. While traditional virus cell culture is considered a relatively slow diagnostic method, sometimes taking weeks for the virus to propagate, modernised methods have allowed the turnaround to be greatly improved (Leland and Ginocchio, 2007).

### Enhanced Virus Isolation Methods

Centrifuge-enhanced shell-vial techniques or well plates, termed cluster plates, facilitate rapid adsorption of the virus into the host cell line by applying low level centrifugation, greatly reducing the length of time required for inoculation of the cell line. In shell vial and cluster plate techniques, the virus infects cells growing in a monolayer, usually on a glass slide, which also facilitates easy analysis of the infected cells under a microscope (Gleaves et al., 1984; Hematian et al., 2016). Another advance in viral cell culture was the development of new cell lines. These include co-cultured cell lines, lines consisting of more than one cell type, and the use of transgenic cells. Co-cultured cells are useful for scenarios where the nature of the virus may be unknown, hence the optimal host is also unknown. Co-cultured lines can save time for diagnostics, as fewer cells lines must be maintained, infected and analysed (George et al., 2002; LaSala et al., 2007). Transgenic cell lines also aid in improved virus diagnosis. Genetically engineered cell lines are modified to contain genes which act as a reporter upon infection with a specific virus. Ideally, this reporter gene will only activate in the presence of virus-specific proteins (Storch, 2000). When using transgenic lines, the virion does not need to be fully assembled for diagnosis to be achieved, as the production of a virus-specific protein is enough to achieve expression of the reporter, thus reducing turnaround time for results (Olivo, 1996). Both co-cultured and transgenic cell lines can be used in conjunction with shell-vial techniques to further reduce time to diagnosis.

### Diagnostic Methods in Virus Isolation

Once growing in cell-culture, there are two categories of methods by which viruses are diagnosed. They consist of those employing observation of the cytopathic effects (CPE) and those which employ molecular methods, known as pre-CPE. Infection of cells with cytopathic viruses causes damage and subsequent morphological changes to the cells which they infect, and these changes are termed CPE. Inspection of the cell cultures under light microscopy is used to determine the presence, or absence of CPE. It can take between 48 h and several weeks for the development of CPE to become apparent. Therefore, this method cannot necessarily provide a rapid turnaround time to results (Fenner et al., 1974). Furthermore, the presumptive nature of CPE-diagnosis means that it may be difficult to discern with confidence which virus is causing the damage. Another challenge is that this method is only applicable to CPE-inducing viruses, and therefore it cannot be considered a universal method for virus detection. To overcome these challenges, molecular methods can be used in conjunction with virus cell culture to provide more accurate and rapid diagnoses. This approach is termed ‘pre-CPE’ diagnosis.

**FIGURE 1:**
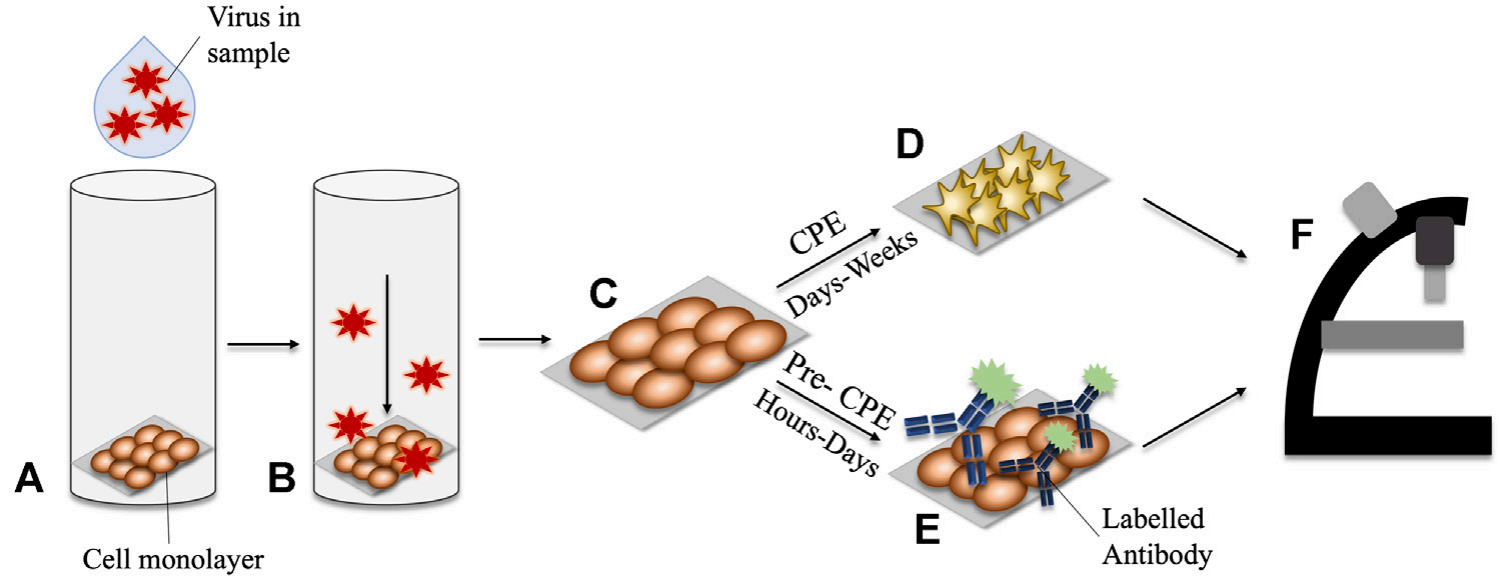
Comparison of CPE vs Pre-CPE detection in shell-vial culture. **(A)** Virus-containing sample is applied to the shell vial. **(B)** A centrifugal force is applied, encouraging inoculation of the virus into the cell monolayer. **(C)** The monolayer cells growing on a glass slide may be removed for interrogation. **(D)** CPE may take days to weeks to develop in the culturing cells. **(E)** Pre-CPE detection employs labelled antibodies to identify virus-specific markers; these can be detected within hours-days. **(F)** Microscopy can be used to inspect for morphological changes in CPE-diagnosis, or for signals arising from the presence of a labelled-antibody for pre-CPE detection.

Pre-CPE methods have the capacity to detect the virus post cell-entry, but before the cells present CPE, meaning they generally provide faster results. Coverslips obtained from shell vial cultures can be used directly for pre-CPE methods. Detection is usually facilitated by staining of the coverslip with an agent which detects a virus-associated protein with high sensitivity and specificity, for example an antibody (Leland and Ginocchio, 2007). An enzymatically- or fluorescently-labelled antibody, is applied to the monolayer of cells on the coverslip. The slip is inspected for a positive signal from the labelled antibody, which in turn indicates a positive confirmation of a certain virus. In general, the use of pre-CPE methods produces comparable results to traditional cell culture methods, with improved turnaround times (Peterson et al., 1988; Rabalais et al., 1992; Lipson et al., 2001). Alongside more rapid turnaround times, pre-CPE detection has the added benefit of employing virus-specific antibodies, meaning that a particular virus, or strain of virus, can be positively confirmed. A schematic of CPE- and pre-CPE-based diagnostic methods is shown in Figure 1.

Although pre-CPE diagnostics prove useful in reducing turnaround times when compared to traditional cell culture, the time to diagnosis can still take days in some cases. In situations where rapid diagnosis is a key factor, for example in cases of extremely damaging or virulent viruses, a turnaround of days is insufficient. While cell culture methods are important for the isolation of viruses, they do not transition well to the current demands for rapid testing. Alongside long result turnaround times, virus culture requires sampling from the viral source and removal to sterile and secure laboratories, where the virus can be grown. In the current climate, where point-of-need/point-of-care testing is highly desired, the steps required for virus culture limit it as a tool for rapid diagnostics. Hence, there has been a shift to molecular techniques which facilitate direct sampling from the source, rapid results turnaround, and amenability for quick adaption to emerging viruses. Techniques which involve the detection of the virus from the source, without any need for virus propagation, fall under the broad category of direct detection.

## Direct Detection

Direct detection methods negate the need for virus propagation. Instead, the virus is detected directly from the suspect source. Direct detection methods usually involve sophisticated techniques, including nucleic acid and immunological detection.

### Nucleic Acid-Based Detection Methods

A key element of nucleic acid-based detection is polymerase chain reaction (PCR) which utilises multiple stepwise temperature cycles, and a polymerase, to amplify DNA strands (Mullis et al., 1986). The standard polymerase used in PCR can only synthesise from a DNA template, thus RNA amplification requires the use of an enzyme with reverse transcription activity (Bustin, 2000). PCR and reverse transcription-PCR are widely applied methods for the detection of both DNA and RNA viruses, respectively (Metcalf, 1995; Pring-Åkerblom et al., 1997; Burkhalter and Savage, 2017; Wadhwa et al., 2017; Liu et al., 2018; Lin et al., 2020). In standard PCR, the DNA products are generally visualised by gel electrophoresis, to check for the presence of expected DNA bands. Using this method, only semi-quantitative results can be achieved, facilitated by running known DNA standards alongside the unknown sample. The method can also fail when samples contain low concentrations of DNA. While high-sensitivity detection can be achieved in some cases, sensitivity can vary greatly depending on the type of DNA stain used, and the type of system used to visualise the gel (Motohashi, 2019). Gel electrophoresis can be used to analyse the results of many alternative nucleic acid amplification methods, such as those based in isothermal amplification, however, the issues remain largely the same. Given that gel electrophoresis can be considered a standard method of analysing amplified DNA products, the following sections will focus more on some of the alternative detection strategies that can be used in post-amplification nucleic acid detection.

#### Real Time Quantitative Polymerase Chain Reaction

**FIGURE 2:**
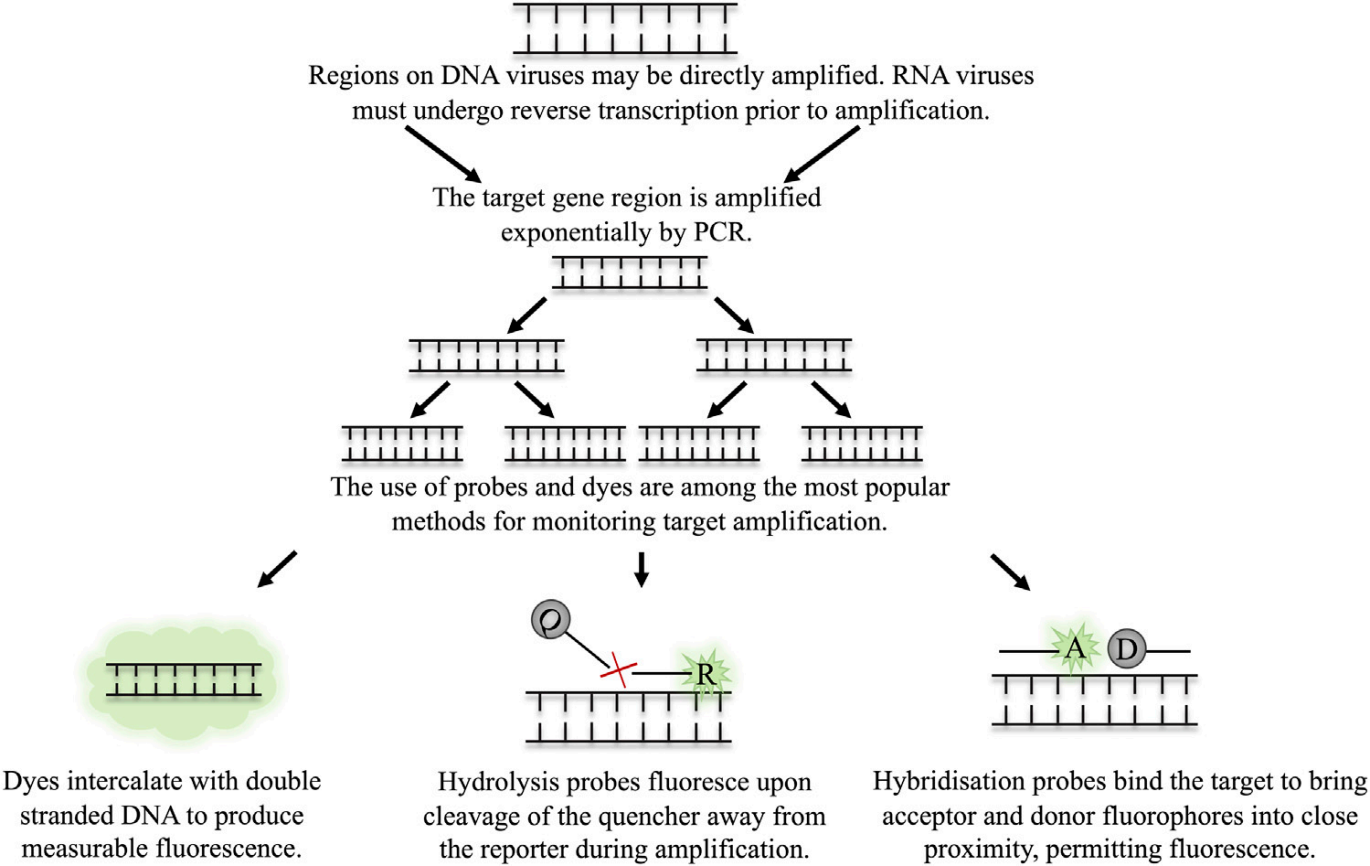
The detection of target amplicon in qPCR through dyes and probes. The target region on the virus is amplified by multiple rounds of exponential amplification in PCR. During amplification, various methods are employed to monitor the real time production of target DNA. **(A)** Dyes intercalate indiscriminately with double stranded DNA, causing an increase in fluorescence as the level of double stranded DNA in the sample increases. Hydrolysis and hybridisation probes require binding to a specific sequence on the target amplicon to permit fluorescence. Fluorescence is achieved either by cleavage of the probe when employing hydrolysis probes **(B)**, or through binding of the probes to the target region in close proximity to one another, as is the case for hybridisation probes **(C)**.

Real time quantitative PCR (real time qPCR) measures the production of the target amplicon throughout the reaction. This is facilitated by DNA intercalating dyes, such as SYBR^®^ green, or fluorescently labelled probes. DNA dyes will bind to all double-stranded DNA. To use these dyes, the primers must be highly optimised and produce no non-specific amplicons as these non-specific products will also produce signal, skewing quantification. Fluorescently labelled probes are a more target-specific alternative. Many types of probe are employed in qPCR, however commonplace probes include those which must bind to a specific region on the target DNA in order for fluorescence to be achieved, for example, hydrolysis or hybridization probes (Navarro et al., 2015). An illustration of the use of fluorescent dyes and probes is depicted in Figure 2. The level of fluorescence in the PCR sample is measured and is directly proportional to the initial concentration of the target in the sample, thus, allowing rapid quantification.

With respect to the development of rapid virus diagnostic assays, real-time qPCR has both benefits and disadvantages. One advantage is the promise of high specificity when using probes such as hydrolysis or hybridization probes, as both the primers and the probe must bind the target sequence to achieve signal. When time-pressure is an issue, an assay which produces highly specific, quantitative results over the course of a few hours is very desirable. While qPCR does offer a relatively quick results turnaround in terms of the assay itself, sample preparation, which is generally required for qPCR, can be a delaying factor. While there are several commercially available kits which facilitate the rapid isolation of DNA or RNA, they come with the disadvantage of added cost (Clark et al., 2016). This, in conjunction with the initial cost of the qPCR equipment itself, could make detection methods like qPCR inaccessible to developing regions. Another desirable trait of a rapid assay is its capacity for high-throughput testing. Given that qPCR is typically used in a 96-well assay format, the transition to high-throughput is feasible (Arya et al., 2005; Eigner et al., 2019). This is of particular interest for situations where high volume testing is vital, for example during pandemics or epidemics, or for industrial or agricultural applications where numerous samples may need to be tested simultaneously. The capacity for qPCR to be performed in multiplexed reactions complements this high-throughput feasibility. The employment of multiple pairs of specific primers and suitable probes, targeting different viruses which may be found in the same sample, facilitates testing for panels of viruses. This, in combination with the multi-well layout of qPCR assays and established high-throughput systems, provides a powerful method to test multiple samples for the presence of several viruses simultaneously (Hwang et al., 2018; Sánchez-Ponce et al., 2018; Eigner et al., 2019; Ou et al., 2020). Although, as aforementioned, the necessary sample preparation may, again, be a limiting factor to the throughput capacity of qPCR assays. This could be overcome by the continued development of novel nucleic extraction approaches such as the magnetic bead-based assays described by Xin and Chen (2012) and Clark et al. (2016). These facilitate high-throughput sample preparation in a short space of time (a few hours) at greatly reduced costs when compared to commercially available high-throughput extraction systems (Xin and Chen, 2012; Clark et al., 2016). Alternatively, sampling strategies such as composite sampling could be employed, whereby a number of samples are mixed and tested as one. The application of composite sampling can achieve higher sensitivity when compared to single sample tests (Jones et al., 2020). This would facilitate multiple sample assessment within a single assay, thereby reducing the level of sample processing required for each run. Furthermore, if one of the combined samples presents a positive result, then further analysis can be performed on single specimens to identify infected individuals. Other drawbacks to qPCR include the high cost of equipment and reagents, the need for strictly controlled temperature cycling, and the necessity for trained staff to run testing. Alongside qPCR, other nucleic acid detection methods are proposed, including those which permit isothermal amplification of DNA and RNA.

#### Loop-Mediation Isothermal Amplification

Loop-mediation isothermal amplification (LAMP), developed by Notomi et al. (2000), relies on the use of multiple primers, at minimum four, to initiate the polymerase-driven extension of the gene sequence. The amplification is facilitated by the formation of stem-loop structures created by the primers. The sequence is then amplified by a strand-displacing polymerase. This is repeated, eventually generating copious amounts of double-stranded DNA product (Notomi et al., 2000; Notomi et al., 2015). The suggested reaction temperature for amplification is 65°C, however, studies demonstrated successful amplification at temperature ranges between 57 and 67°C (Tomita et al., 2008; Francois et al., 2011). The reaction time typically ranges from 30 to 60 mins, although with optimisation of the reaction conditions, such as reaction temperature and buffer composition, it was shown to be possible to reduce the time to result to 10 mins (Tomita et al., 2008; Estrela et al., 2019). This time can be reduced by the introduction of another set of primers, termed loop primers (Nagamine et al., 2002). Additionally, swarm and stem primers can be introduced to the reaction to further reduce reaction duration (Gandelman et al., 2011; Martineau et al., 2017). Where the target is RNA-based, a reverse transcriptase can be incorporated directly into the LAMP reaction, termed RT-LAMP (Wong et al., 2018). The singular assay detection of both DNA and RNA is appealing as it reduces the “hands-on” time for target amplification, with particular note to RNA-based viruses.

The resulting amplicons from LAMP are detected through a number of means. Intercalating dyes, such as SYBR green can be added either to the end-point reaction, allowing for a rapid qualitative result, or added prior to the reaction to measure the real-time increase in fluorescence (Zhang X. et al., 2014). Some dyes may have inhibitory effects on the LAMP reaction, hence, a range of dyes should be assessed in order to identify the most suitable for each assay (Quyen et al., 2019). Fluorescently labelled probes can also be employed. However, probes developed for qPCR may be unsuitable for LAMP due to the strand displacing activity of the polymerase. To overcome this, alternative probe-based methods are proposed, including quenching of unincorporated amplification signal reporters (QUASR), detection of amplification by release of quenching (DARQ) or mediator displacement probes (Tanner et al., 2012; Ball et al., 2016; Becherer et al., 2018). Gold nanoparticles (AuNPs)-labelled with DNA, complementary to a sequence on the target, are proposed as another option for amplicon detection (Arunrut et al., 2016). Currently, there is no single optimised way to incorporate probes into the LAMP reaction and further work is required to standardise the probe-based target detection. The turbidity of the reaction is also used for both qualitative and quantitative analysis. LAMP produces large amounts of precipitated magnesium pyrophosphate as a by-product. Typically, the presence of precipitate is visible to the naked eye after a successful reaction, allowing a rapid qualitative answer (Zhang et al., 2014). Turbidity within the assay can also be measured real-time via the employment of a turbidimeter, which facilitates quantitative analysis of target DNA by comparing an unknown sample to a standard curve (Parida et al., 2008; Wang et al., 2020).

**FIGURE 3:**
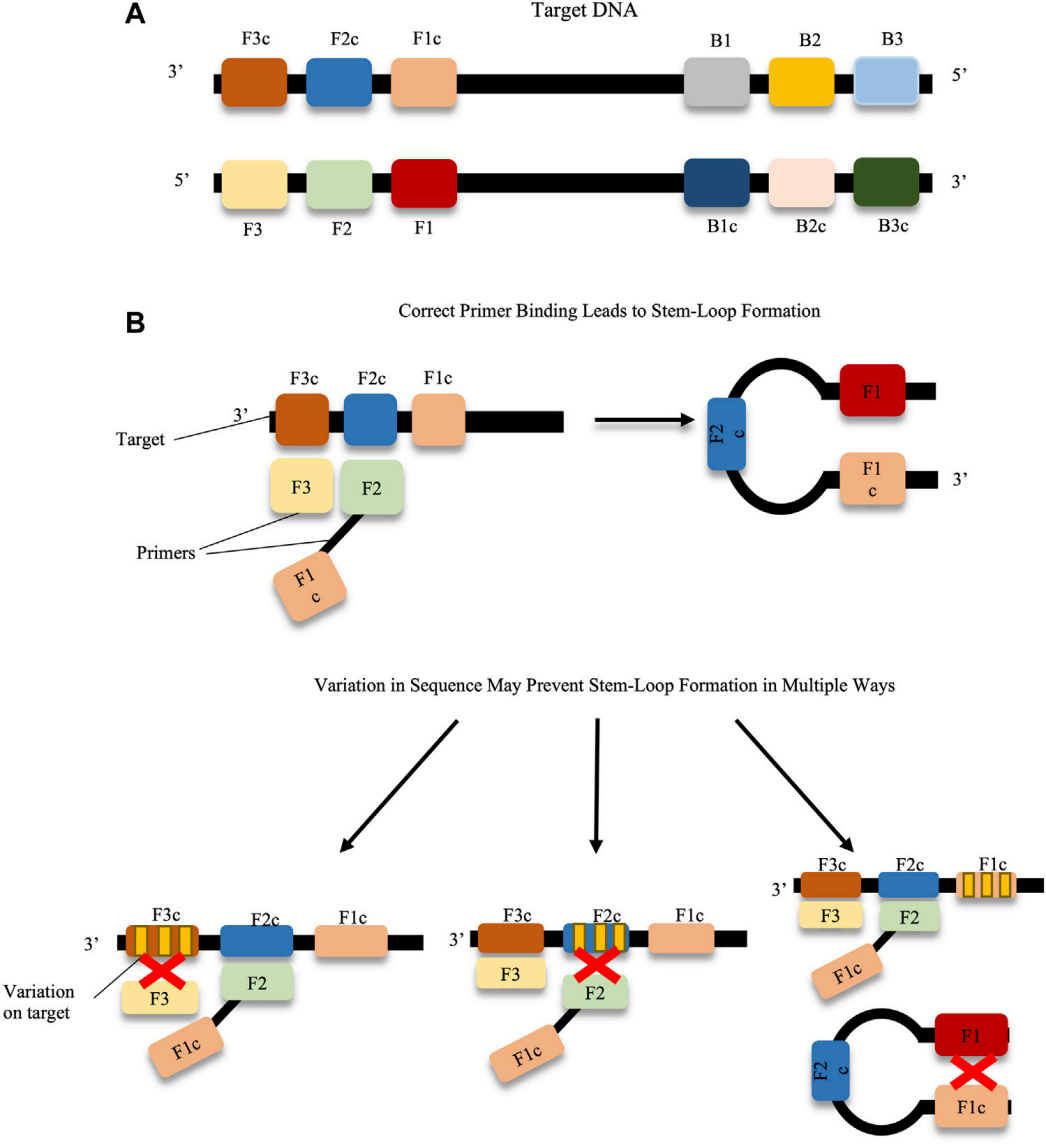
Prevention of stem-loop formation caused by target sequence variations. **(A)** F1-F3, F1c-F3c, B1-B3 and B1c-B3c on the target DNA represent the binding regions for the LAMP primers, e.g. primers F3 and F2-F1c as shown in the diagram. If each of the primers matches suitably with the target DNA, stem loop formation proceeds. **(B)** Variation across any primer-binding sites (represented by yellow bands on target regions) can prevent stem-loop formation, and hence amplification. Primer design can be challenging when trying to avoid these variable regions.

With respect to virology, LAMP offers a highly sensitive and rapid alternative to classic PCR, or qPCR. Amplification by this method does not require sophisticated apparatus as the reaction is maintained at a single temperature, typically around 65°C (Tomita et al., 2008). This temperature, however, does mean that some heating apparatus is required. This may be overcome by developments such as the employment of phosphorothioated primers, and additives such as single-stranded binding proteins and urea, which permit the performance of the LAMP assay at 40°C, albeit with a longer reaction time than was observed with traditional LAMP (Cai et al., 2018). LAMP was also shown to be robust when detecting directly from sources such as blood, urine, stool various media components and insects, meaning there was no need for additional DNA/RNA extraction equipment (Kaneko et al., 2007; Francois et al., 2011; Silva et al., 2019). As LAMP employs multiple primers the method is very specific. In order for the assay to function, the primers must bind to six regions on the sequence. However, the primers must be designed to have specific distances between their target regions, while only amplifying a product of maximum 300 bp. This can make the design of LAMP primers complicated, even with the available software to aid in their design (Parida et al., 2008; PrimerExplorer, 2020). The challenges faced with primer design may be further exacerbated by the variation in viral gene sequences across different strains, which may make the identification of conserved and suitable primer-binding sites difficult (Parida et al., 2008; Sanjuán and Domingo-Calap, 2016; Duffy, 2018). An illustration of potential issues faced with primer-binding and sequence variation is depicted in Figure 3. When validating LAMP assays, care should be taken to ensure that all strains of a virus are amplified equally with the primer set, and that no false negatives are observed.

Another challenge faced by LAMP is carryover contamination. While contamination poses a threat to many amplification methods, LAMP is particular prone to the effects due to its high sensitivity. Contamination is usually caused by products from previous experiments carrying over via the environment, researchers clothing, or lab equipment. The contaminant products and are then used as templates in new reactions, causing false positives in some cases (Dhama et al., 2014; Hsieh et al., 2014). This can be mitigated by avoiding results analysis which requires the opening of reactions tubes, or by using alternative methods which incorporate dUTP, in lieu of dTTP, in the LAMP reaction. The dUTP is cleaved by uracil-DNA glycosylase prior to the next LAMP experiment, thus preventing previous amplicons from acting as templates in subsequent reactions (He and Xu, 2011; Hsieh, 2014). In a situation where the virus being tested is harm-causing, the likelihood of the outcome being a false-positive should be as low as possible to avoid the administering the wrong treatment or negative outcomes for patients (Borst et al., 2004). Further to this, in a situation of rapid viral spread such as pandemic or epidemic, a high false positive rate may put pressure on healthcare systems if tests have to be performed multiple times to ensure accuracy.

#### Recombinase Polymerase Amplification

RPA was proposed by Piepenburg et al., in 2006, and since then has gained traction as a promising method for nucleic acid detection (Piepenburg et al., 2006). RPA introduces two proteins, a recombinase and a single-stranded DNA binding protein (SSBP), in the presence of other reagents such as a strand-displacing DNA polymerase and 30–35 bp long, target-specific oligonucleotides pairs (primers). RPA is initiated by the formation of a complex between the oligonucleotides and the recombinase. The complex scans double stranded DNA for sites complementary to the primers. Once target sites are identified, the complex invades the double stranded DNA, the displaced strand is stabilised by SSBPs and the recombinase dissociates. Exponential amplification is achieved through repeated cycles of this process. The RPA reaction has an extremely flexible reaction temperature, typically between 22 and 45°C. Further to this, the reaction is usually complete within 20 min (Piepenburg et al., 2006; Daher et al., 2016; Lobato and O’Sullivan, 2018). Amplification of RNA can be achieved via the addition of a reverse transcriptase directly to the RPA mixture, making the reaction time-friendly for RNA-detection (Euler et al., 2012; Daher et al., 2016).

Upon development of the RPA assay and subsequent amplification of the target, there are multiple ways in which the resulting amplicon can be detected. For quantitative, real-time results, fluorescent probes can be used, similar to qPCR. These probes aid in increasing the specificity of the technique, as only probes which bind to a complementary DNA sequence will fluoresce. Currently, RPA probes, termed exo and fpg, both require the cleavage to separate the reporter fluorophore from the quencher (Euler et al., 2013; Yang et al., 2015). Probes which facilitate detection of amplicons through lateral flow technology are also available. Detection through this means is usually in sandwich assay format and is termed nucleic acid lateral flow immunoassay (NAFLIA). In this process, throughout amplification, the amplicon is labelled with two different “antigenic” labels. These labels are then captured by label-specific antibodies on the lateral flow strip (Powell et al., 2018). The fact that RPA offers both quantitative real time detection via the use of fluorescent probes, and qualitative, or semi-quantitative, detection via agarose gel detection or NALFIA makes it appealing to the field of virology.

RPA has a quick turnaround time and provides high flexibility in terms of reaction temperature. When envisioning assays for viral detection, as with all nucleic acid amplification methods, care should be taken with primer design. However, this may be particularly relevant for RPA due to the fact that it operates at a lower temperature, which can reduce specificity (Wu et al., 1991; Luo et al., 2019). A study performed to investigate the effect of base pair mismatches between primer and target sequence found that while the RPA reaction was tolerant to some mismatches, the tolerance changed depending on the type of mismatch. For example, mismatches between bases near the 3′ end of forward and reverse primers had a larger negative impact than those elsewhere on the primers. They also showed that RPA efficiency was further reduced in situations where the mismatched sequences contained either very high or very low GC content (Daher et al., 2015). The implications of this for virus detection is that caution must be taken when choosing a target region in the viral DNA or RNA. It is established that the mutation rates and variation in viral gene sequence can be high (Sanjuán and Domingo-Calap, 2016; Duffy, 2018). While variations across target sequences can be useful in differentiating between strains, it poses a challenge if the overall goal is to detect all strains of a virus indiscriminately. For RPA it is important to ensure that there is no amplification bias between strains. Further to this, given that RPA exhibits tolerance toward mismatches, extensive analysis into any potential cross-reactive non-target sequences must be performed. Hence, when choosing a target sequence to detect, the optimal choice is one that is relatively conserved across the virus to be detected but does not show high sequence similarity to other non-target sequences. While the lower temperature reaction means that added care must be taken in primer design, it offers the benefit of a reduced equipment demands, as typically only a simple heating instrument is required. The reactions are robust to changes in temperature, allowing flexibility in situations where on-site temperature changes may cause variation. This flexibility was demonstrated by Lillis et al. (2014) where they performed successful RPA reactions at ambient air temperatures above 31°C, in the absence of any additional heating equipment. They also proposed the use of exothermic chemical heaters, such as sodium acetate trihydrate (SAT) heaters. SAT is a re-useable chemical which releases heat upon crystallisation. Chemical heaters such as these are commonplace in hand-held heaters, making this technology widely available at relatively low cost. The group demonstrated successful RPA at ambient temperatures of 10°C when the reaction was warmed with SAT-based heaters (Lillis et al., 2014). In a similar manner, successful RPA reactions were even achieved using body heat alone, with no additional heating equipment (Crannell et al., 2014). The reduced need for heating also lowers the overall cost of detection, making this technology more accessible to areas with reduced resource availability (Londoño et al., 2016). Another factor which reduces cost, and time associated with assay development, is that there is no need for RNA or DNA extraction from the viral sample. RPA functions well using crude sources, including urine and blood, albeit with a slight reduction in the sensitivity of the assay in some cases (Clancy et al., 2015; Rosser et al., 2015).

#### Helicase-dependent Amplification

Helicase-dependant amplification (HDA) functions by employing a DNA-helicase to unwind double stranded DNA. Flanking primers anneal to the strands, where a polymerase amplifies the target region. The process is repeated to exponentially amplify the target. When initially developed, HDA was performed at 37°C and required additional proteins such as SSBPs and MutL for the amplification to commence. However, further development of the reaction has negated the need for the SSBPs and MutL. These developments also incorporate a thermo-stable helicase and polymerase, permitting the reaction to be performed at higher temperatures between 60 and 65°C (Vincent et al., 2004; An et al., 2005; Teo et al., 2015). As with RPA, the primers are longer than typically used in PCR, reaching around 30 bp for HDA. To facilitate the direct amplification of RNA viruses, a RT enzyme can be added directly to the HDA reaction in a one-step protocol (Goldmeyer et al., 2007).

The amplified products can be detected through the use of fluorescent dyes and lateral flow-based detection (Chow et al., 2008; Kim et al., 2011; Li et al., 2013; Kolm et al., 2019). As HDA employs a similar mechanism to the standard PCR reaction, with the main difference being the helicase-mediated separation of DNA rather than heat-separation, molecular probes commonly used in qPCR could also apply to HDA. Tong et al. (2008) demonstrated the application of molecular probes in conjunction with HDA to facilitate probe-based quantitative diagnostics. The fact that similar probes can be used, indicates that a transition from assays such as qPCR to HDA may be relatively easy, while at the same time providing a low-cost, isothermal alternative to traditional PCR.

As HDA only requires two primers and a small number of proteins to function, it offers a more simplistic assay design when compared to other isothermal reaction, such as LAMP. This could appeal to researchers who wish to rapidly establish diagnostic tests. Further to this, the ability to repurpose already established qPCR molecular probes would also hasten the time in which a HDA assay could be developed. However, this simplicity of design does come at the cost of reduced specificity, as issues with non-specific primer annealing can occur with HDA (Barreda-García et al., 2018). Non-specific amplification could be overcome in part by the application of molecular probes as these provide an added specificity requirement. As with other isothermal reactions, only simple heating apparatus is required, however, the higher temperature reaction of HDA negates the ability to use ambient temperatures for amplification. HDA functions in the presence of other biological agents, and can be used alongside crude samples, without the need for DNA or RNA extraction (Kim et al., 2011; Li et al., 2013; Jevšnik et al., 2020).

#### Rolling Circle Amplification

**FIGURE 4:**
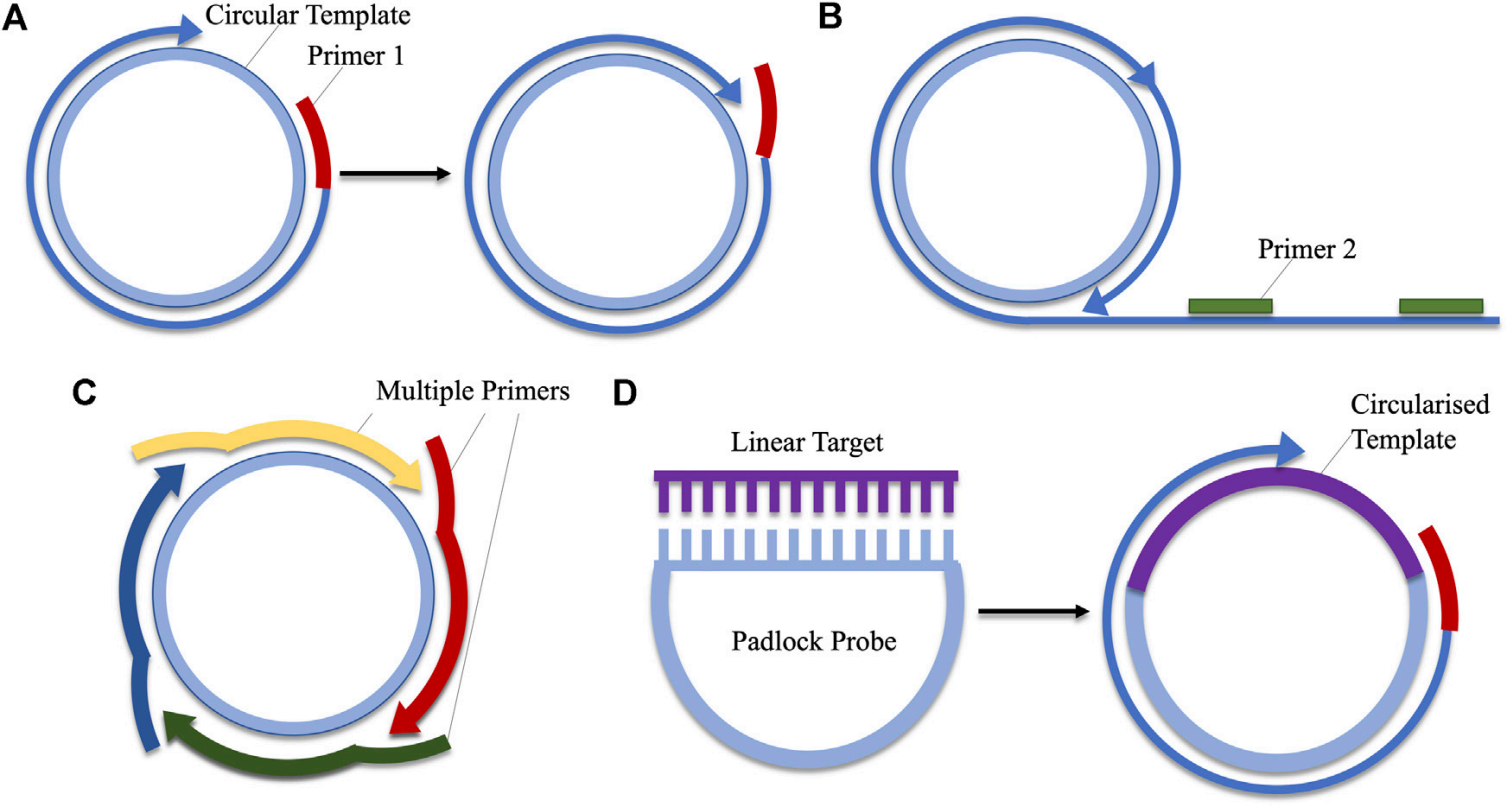
A comparison of the various types of rolling circle amplification. **(A)** Linear amplification using a single primer. **(B)** Hyper-branched RCA adds a second primer which aids in exponential amplification by binding to regions on the repeated DNA sequence. **(C)** Multiply-primed RCA also facilitates the production of large amounts of amplicon via the use of several primers which bind to different regions on the circular DNA. **(D)** Padlocks probes contain sequences complementary to linear DNA. Hybridisation of these regions facilitates ligation, producing a circularised template which can be employed in RCA.

Rolling circle amplification (RCA) mimics a natural process by which circular DNA is replicated (Kasamatsu and Vinograd, 1974; Mohsen and Kool, 2016). This process is adapted for use in the amplification of circular DNA sequences, making it unique from the previously described methods which amplify linear DNA. At its most basic, RCA requires only one primer, from which a strand displacing polymerase initiates replication. The resulting amplicon is a linear product, consisting of repeat sequences of the circular DNA (Schweitzer and Kingsmore, 2001; Mohsen and Kool, 2016). This method of RCA does not facilitate exponential amplification, however, alterations may be made to the protocol to facilitate rapid, exponential amplification. These include the use of hyper-branched RCA, which utilises two primers. The first binds to the circular target-DNA, while the second is complementary for a separate region on the single-stranded product. As the circular DNA is replicated multiple times, this second primer continuously binds repeat regions, creating a branched amplicon (Lizardi et al., 1998; Hamidi et al., 2015b; Li et al., 2019). Multiply-primed RCA is another alternative which uses several primers. Each primer anneals to a specific region on the circular DNA template and elongation is initiated. Amplified strands are displaced by the polymerase, and secondary primer-binding can occur on the displaced strands, creating large quantities of amplified product (Dean et al., 2001; Marincevic-Zuniga et al., 2012; Fux et al., 2018). Another important variation of RCA is padlock probe-based RCA. As RCA can only act upon circularised products, these probes are used when the desired target is single-stranded, linear DNA. The padlock probe features two regions, complementary to two regions on target. Upon hybridisation of the probe and the target, the two are joined via a ligase-mediated reaction, creating a circular template on which RCA can act (Banér et al., 1998, Banér et al., 2001; Mezger et al., 2014). The RCA reaction can be performed at a range of temperatures between 30 and 65°C (Marincevic-Zuniga et al., 2012; Li et al., 2019). The various forms of RCA are compared in Figure 4.

The resulting products can be detected in real-time through the use of intercalating dyes (Kaocharoen et al., 2008). Fluorescently labelled probes can also be used to label the amplified product (Mezger et al., 2014). Real-time detection is facilitated through the use of molecular beacons (Nilsson et al., 2002). Product formation may also be measured through colorimetric means, using hydroxy naphthol blue (Hamidi and Ghourchian, 2015). Lateral flow technology can also be used to detect labelled, amplified products (Liu et al., 2019).

RCA is a rapid, sensitive and specific technique, useful for application to viral diagnostics. The assay design for RCA is relatively simple, requiring no target denaturation, and at minimum needing only one primer to amplify circular DNA or RNA (Li and Macdonald, 2015). However, this linear amplification method does not produce a high yield of product, therefore exponential RCA methods are typically used. With regards to the amplification of linear targets, for example single or double stranded linear viruses, padlock probes are useful. However, in order to perform this reaction, the target must first be denatured, and subsequently hybridised and ligated to the padlock probe. This adds to the length of time until diagnosis and the hands-on time is also increased. As RCA can take as little as 30 min, and requires minimal hands-on time, any additional reaction steps detract from the appeal of RCA (Kaocharoen et al., 2008; Mezger et al., 2014). This also contrasts to other methods such as RPA and HDA, which do not require preliminary denaturation steps. Nonetheless, there remain advantages for the use of padlock probes in viral detection. For example, the high specificity required to achieve both target hybridization and ligation means that non-specific binding is less likely to occur and produce false positives. It was also shown that multiple padlock probes, featuring varying hybridization sequences, used in the reaction, did not result in hinderance of the target-probe ligation process. This is useful for the detection of viruses which may have high levels of variation across the target region as all variants can be detected in a single reaction (Mezger et al., 2014). Alongside viral diagnostics, RCA can also be used for rapid viral genome amplification, facilitating sequencing of emergent strains, or comparisons between variants (Zanoli and Spoto, 2013). Therefore, it appeals to a number of streams within the virology field.

#### CRISPR-Cas-Based Nucleic Acid Detection

**FIGURE 5:**
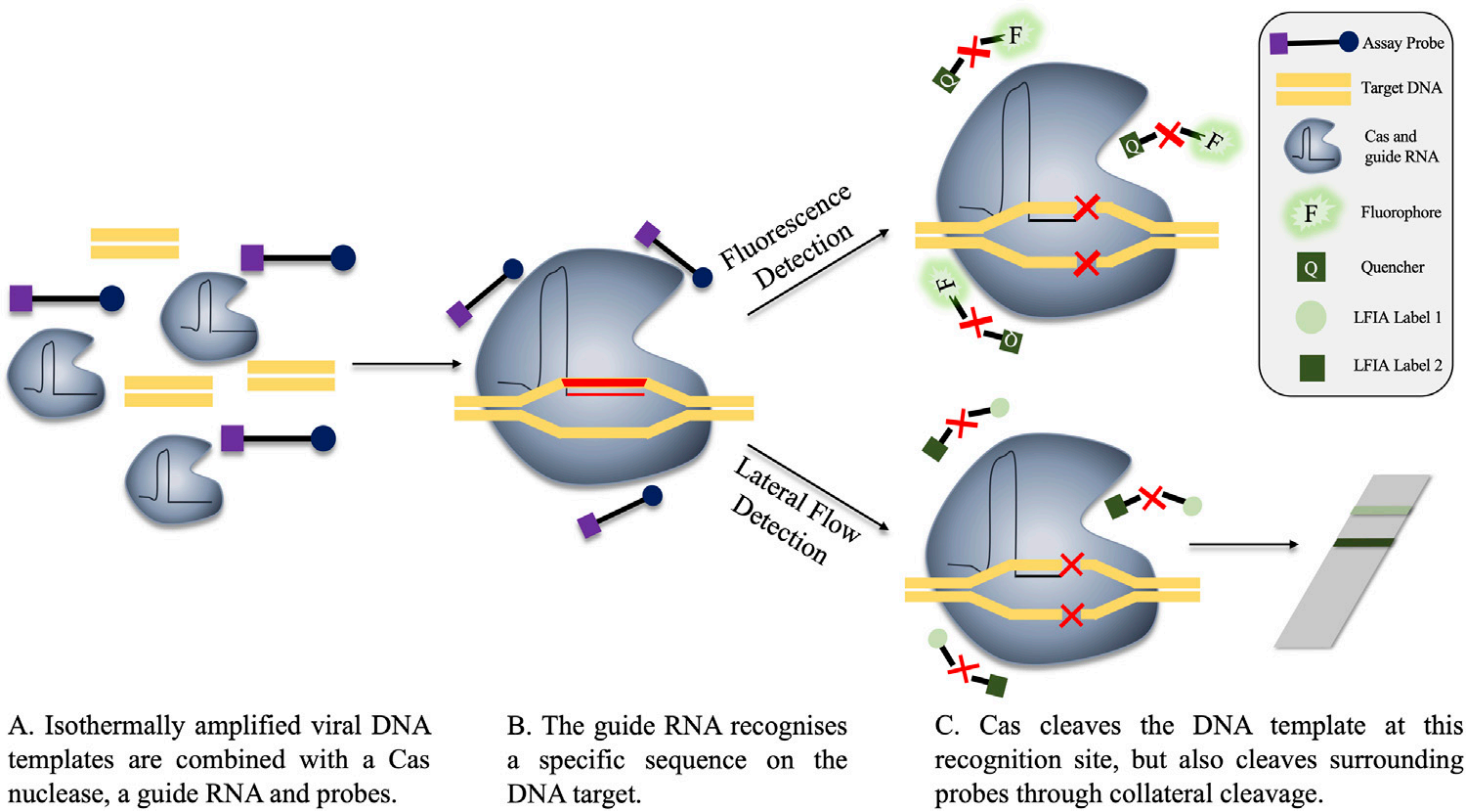
Fluorescence and lateral flow-based detection in a CRISPR-Cas detection system. **(A)** The components of the reaction are combined, either before or after isothermal amplification. The guide RNA is specific for a region on the target viral DNA. **(B)** The guide RNA recognises the target sequence, locating the Cas nuclease to this region. **(C)** The DNA target is cleaved by the Cas nuclease mechanism. However, Cas also performs collateral cleavage on surrounding single-stranded probes. Cleavage is represented in the diagram by a red X. These probes can be fluorescent (**C**, top), where the cleavage of the probe separates the quencher and the fluorophore, permitting fluorescent signal. Alternatively, probes can be used in lateral flow assays, whereby cleavage of the probe produces two distinct bands on the flow-strip, indicating a positive result. A single band on the lateral flow strip is representative of a negative result as no cleavage events occurred and the labels are not separated (**C**, bottom).

Cas proteins are effector proteins which play an integral role in the clustered regularly interspaced short palindromic repeats (CRISPR)-Cas system. Cas effector proteins have the ability to cleave DNA or RNA. Their cleavage activity is controlled by guide-RNAs, which direct the Cas proteins to the correct cutting site on the target nucleic acid. The guide RNA of the CRISPR-Cas system can be modified to facilitate targeting of specific sequences, and herein lies the application to nucleic acid detection (Bhattacharyya et al., 2018). Three primary systems are thus far proposed for nucleic acid detection, named SHERLOCK (specific high-sensitivity enzymatic reporter unlocking), DETECTR (DNA Endonuclease Targeted CRISPR Trans Reporter) and HOLMES (one-HOur Low-cost Multipurpose highly Efficient System). SHERLOCK uses Cas13a to detect RNA, while HOLMES and DETECTR use Cas12a for DNA recognition (Gootenberg et al., 2017; Chen et al., 2018; Li et al., 2018). As aforementioned, the Cas nuclease cleaves the DNA or RNA at the recognition site, however, off-target cleavage can also occur with some Cas proteins. This is known as collateral cleavage. While this is a naturally occurring mechanism, collateral cleavage can be repurposed for nucleic acid detection. Normally to facilitate Cas-mediated nucleic acid detection, the target nucleic acid is first amplified via an isothermal reaction, usually RPA. Thereafter, the CRISPR-Cas system is introduced, featuring a guide RNA specific for a location on the target DNA. Binding of the guide RNA induces target cleavage, but collateral cleavage also occurs. Measurable signal in the assay can be achieved by the addition of fluorophore probes, which are cleaved by the collateral activity of the DNA-recognising Cas protein, Cas12a, resulting in measurable fluorescence (Batista and Pacheco, 2018). The reaction can be performed in a “one-pot” style, with results achieved in 1 h (Li et al., 2018; Kellner et al., 2019). The amplified and cleaved products can be detected through fluorescence measurement, or, for a simpler alternative, on a lateral flow strip (Gootenberg et al., 2018; Myhrvold et al., 2018; Broughton et al., 2019). An overview of both fluorescence- and lateral flow-based CRISPR-Cas detection is depicted in Figure 5.

The dual use of isothermal amplification and CRISPR-Cas proteins provides a rapid assay with an added specificity safety net. First, primer sets must bind to the target to facilitate initial product amplification, then the guide RNA must detect a specific sequence on this product to facilitate signal generation. This dual mechanism could help alleviate issues associated with the non-specific primer binding sometimes observed in isothermal amplification. The fact that both amplification and Cas-cleavage can be performed in a single reaction is also appealing, as it reduces the risk of contamination and requires less hands-on time. Given that already existing isothermal amplification methods can be transitioned to CRISPR-Cas nucleic acid detection, these enhanced assays could be rapidly developed against a host of viruses. It is likely that with the improved specificity that this detection system offers, that its application will continue to grow in the field of virology.

#### Automated Systems for Nucleic Acid Detection

The capability for high-throughput and/or multiplexed detection of viruses in a sample is an important factor to consider when choosing a suitable diagnostic method. Automated systems based on the amplification of nucleic acids from a sample are, therefore, an extremely useful tool in scenarios where multiple samples require assessment, or where a sample must be tested for the presence of a range of viruses. Currently, several systems are available for the high-throughput sensitive detection of nucleic acids, examples of which include the BioFire FilmArray, Verigene, Gen-Probe Prodesse and Cepheid systems (Babady, 2013; Van Wesenbeeck et al., 2013). Such systems are capable of running multiplexed diagnostic assays, with the number of samples used per run varying depending on the platform. Results turnaround is generally within a day, with some systems capable of rapid testing, producing results within an hour. In addition to a rapid run time, integrated sample preparation is also available on several systems, reducing hands-on time to as little as several minutes (Babady, 2013; Van Wesenbeeck et al., 2013; Huang et al., 2018). The offer of contained sample preparation within some automated systems is advantageous when the sample may contain a high-risk viral pathogen (Loftis et al., 2017). When large-scale viral detection is required, automated systems may offer an advantage over amplification methods such as isothermal methods due to their amenability to multiplexing and high-throughput analysis. However, the requirement for specialised equipment may limit the suitability of these automated assays in low resource settings. Nonetheless, the capacity to assay multiple samples simultaneously is highly useful for application in settings where large-scale testing is necessitated.

In summation, methods which rely on nucleic acid detection are useful tools for analysing viral infection. They are often very sensitive and specific, ranging in complexity of design from LAMP, which requires at least four primers to function, to RCA which at its minimum only requires one primer. The higher the number of oligonucleotides which must bind to the target for the assay to proceed generally means a lower rate of non-specific binding and non-target product formation. While qPCR is still in commonplace use in laboratory-based situations, isothermal techniques are witnessing a surge for applications such as point-of-care/use and in low-resource settings. Furthermore, many isothermal amplifications are relatively robust when it comes to the nature of the sample from which the target DNA/RNA is amplified. This is, again, appealing for on-site diagnostics but also for scenarios where time is of the essence and avoiding sample preparation would reduce the time to result. In addition to qPCR and isothermal techniques, automated DNA amplification-based platforms are an important tool in viral diagnostics as they generally offer quick turnaround times and require limited manipulation of samples. Another strength of nucleic acid-based detection systems is the speed at which they can be designed when the sequence of the virus of interest is available. Given that viruses can rapidly emerge, the ability to quickly develop assays to detect particularly damaging, pathogenic or transmissible viruses is of major interest. Therefore, nucleic acid techniques will remain essential in the future of virology. Nonetheless, there are other methods which are important for virus detection. These include methods which employ biological detection agents, such as antibodies, to identify virus-associated proteins.

### Immunoassay-Based Viral Diagnostics

Immunoassays employ antibodies as the primary means to detect viruses within a sample. Antibodies are available in polyclonal, monoclonal and recombinant formats (Ma and O’Kennedy, 2015). Polyclonal antibodies bind multiple different structures or epitopes on the target organism/molecule i.e., are polyspecific. This can be useful in situations where the desired outcome is indiscriminate detection of all viral strains, or when the goal is to capture all strains of a virus from a sample. In contrast, monoclonal antibodies, and certain recombinant forms of antibodies, present singular-epitope specificity, hence, they are monospecific. Specific antibodies are valued in diagnostics as they offer the opportunity for the targeted detection of distinct regions on the target molecule. In virus diagnosis, this is useful for differentiation between different isolates or genotypes of a single virus, or between similar viral species within a genus (Usuda et al., 2000; Yu et al., 2005; Chen et al., 2016; Lebani et al., 2017).

When the targets are highly similar, for example similar strains of a given virus, isolating non-cross-reactive antibodies can be challenging. To overcome this, antibodies can be isolated based on their ability to detect short peptide sequences. These peptides correspond to unique regions on the target protein, providing the capacity to detect strain-specific regions on an entire protein (Geysen et al., 1985). When employing this strategy, it is essential to carefully select the target peptide. Peptides can sometimes adopt conformations which do not reflect the epitope in its native conformation. Given that antibody binding is affected by both amino-acid sequence, and by structural conformation of the amino acids in the binding regions, ensuring comparable binding is essential (O’Kennedy et al., 2017). Therefore, the binding to both the peptide and the native epitope on the target protein must be validated (Conroy et al., 2009). Overall, targeting highly specific peptides, rather than an entire protein with multiple epitope regions, could result in antibodies which are less likely to cross-react with other viruses or isolates of the viral species.

The initial development of pathogen-targeting antibodies requires time to identify a valid disease-specific antigen, isolate suitable antibodies and validate the resulting antibodies and assays. However, once generated, the production of the antibody proteins is relatively fast. Furthermore, the advent of recombinant antibody technology, whereby antibodies are produced as fragments of the whole antibody protein, has made the generation of antibodies more rapid and cost-effective, as cheaper expression hosts such as yeasts and bacteria can be used in lieu of mammalian cell-culture (Jeong et al., 2011; Kunert and Reinhart, 2016).

Virus-targeting antibodies are used in virology for multiple purposes. Two major applications are for the detection of virus-associated antigens from samples, and for determining seroprevalence. Seroprevalence assays are essential techniques for identifying patients who have been exposed to a virus (Li et al., 2020). However, these assays rely on the identification of anti-virus antibodies generated by the host, whereas this review focuses on the creation, employment and design of specific antibodies to detection pathogen-associated targets. As such, the following section largely deals with the use of antibodies for identifying virus-associated antigens from samples, with only brief mention to applications for seroprevalence monitoring. The detection of virus-antigens was initially facilitated by virus propagation through cell culture methods, whereby antibodies are used to detect virus-associated proteins within a cell line, or to perform virus neutralisation assays (Leland and Ginocchio, 2007). However, as discussed, such cell culture methods are relatively slow when compared to other diagnostic means. Therefore, much focus is on the development of antibody-based assays capable of detecting the target-virus directly from source through a range of antibody-based assays, termed immunoassays.

#### Enzyme-Linked Immunosorbent Assay

**FIGURE 6:**
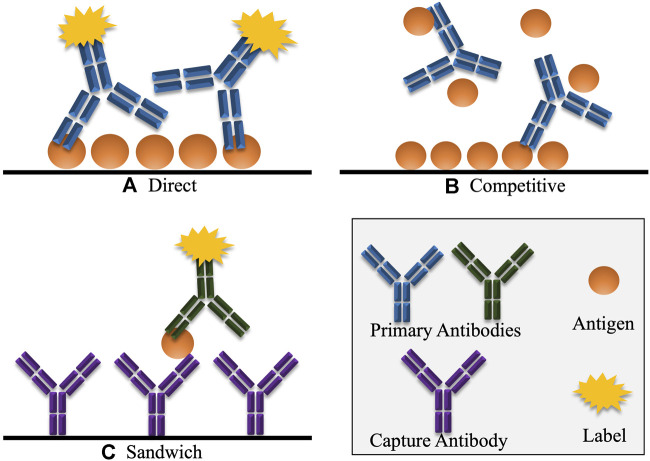
Various common formats of immunoassays, including ELISA. **(A)** In direct detection, the antigen is detected directly by a labelled antibody. Indirect detection is a variation of this format, where the primary antigen-specific antibody is unlabelled, and must be detected by a secondary labelled antibody that selectively binds to the primary antibody. **(B)** In competitive detection, free antigen in the sample competes with immobilised antigen for binding to the antibody which may be directly labelled or may be detected using a secondary labelled antibody. Hence, in competitive immunoassays, the signal is inversely proportional to the concentration of antigen. **(C)** In the sandwich immunoassay format, the antigen is captured by an antibody that reacts with a specific epitope on the antigen. A second labelled antibody is added which reacts with another different epitope on the captured antigen. Here the signal generated is directly proportional to the amount of antigen present.

One of the most widely applied immunoassays is the enzyme-linked immunosorbent assay (ELISA). There are a number of formats in which the ELISA assay can be performed, namely direct, indirect, competitive and sandwich (Figure 6). These formats are also used for most other immunoassays.

Direct and indirect formats are similar. Initially, the antigen is coated to the surface of the ELISA well through passive adsorption, or it may be chemically linked. For direct detection, a labelled, anti-target primary antibody is applied to the well, and subsequently detected. For indirect detection, the primary antibody is unlabelled, and is detected by the addition of a labelled, secondary antibody. These formats are useful for measuring an antibody response against a given antigen. For example, with regards to virology, direct or indirect ELISA assays are useful for confirming patients who have anti-virus antibodies in their sera, having developed an immune response post-viral infection (Khudyakov and Kamili, 2011; Bréard et al., 2013; Zhang et al., 2020). However, there are some drawbacks to direct and indirect formats when transitioning to detecting viruses in crude samples such as blood, stool or other tissues. For both formats, the suspect sample may first have to be coated onto the ELISA test well. Most biological samples will contain a combination of target proteins and other proteins. These other proteins compete for adsorption to the assay well surface, meaning that the target-antigen may not be accurately represented in the sample, making quantitation challenging (He, 2013). To overcome this, formats such as competitive and sandwich are used.

Competitive assays are effective in the detection of viral-specific proteins from a given source. Competitive ELISA first involves the immobilisation of the target antigen to an ELISA well, followed by the simultaneous application of the antibody and the suspect sample to the same well. Target-antigen in the sample competes for antibody-binding, leaving less antibody available for binding to the immobilised antigen. The sample is washed away, and any remaining bound antibody is detected with a labelled, secondary antibody. Therefore, in this assay format, the signal is inversely proportional to the amount of analyte in the sample. Competitive analysis is widely used for the detection of small antigens, where the binding of multiple antibodies may not be permitted (He, 2013). This assay format is useful when considering the rapid-development of viral-detection assays as it only requires one target-specific antibody. However, the use of only one antibody also has consequences in the form of reduced assay sensitivity and specificity if any cross-reactivity with the sample matrix occurs.

The employment of a sandwich formats overcomes most issues associated with cross-reactivity as it employs two antibodies, both specific for different regions (epitopes) on the target molecule. The first of these is immobilised to the well surface. Thereafter, the sample is applied, and specific antigen is captured by the immobilised antibody. Unbound entities are washed away, and the second antibody is applied. This second antibody can be labelled directly or detected with a labelled-secondary antibody. This format offers high specificity as it requires the binding of two antibodies to produce a signal. This could be helpful for virus detection where there is a risk of cross-reactivity occurring between similar strains of virus. It is also considered the most sensitive and robust ELISA format (Ecker et al., 2013; He, 2013). Sandwich assays are less prone to cross-reactivity. Even if one of the antibodies in the pair has some cross-reactivity with the sample matrix, the likelihood is that the same cross-reactivity will not be observed with the alternate binding antibody. This makes this assay suitable for the detection of viruses directly from their sources, such as serum or tissue samples, where the matrix can be very complex (Jansen van Vuren and Paweska, 2009; Zhang et al., 2018). A drawback to the sandwich assays is the complexity of design. Both antibodies need to bind, unhindered by one another, to the target molecule. Finding a suitable binding pair can be difficult, and thorough assay validation is required to ensure no non-specific interaction occurs between the antibodies within the assay. However, the use of antibody-discovery technology, such as phage display, has led to the development of methods which facilitate the identification of binding pairs of antibodies in a rapid manner, negating the time-consuming screening associated with finding two separate binding pairs (Gorman et al., 2017). Alternatively, the previously described strategy of peptide-targeting can be employed to design antibodies which can detect distinct regions on the whole antigen molecule, facilitating sandwich detection.

ELISA continues to remain a staple platform in virus detection due to its sensitivity and robustness. However, there are drawbacks to its use. These include the risk of cross-reactivity of antibodies to other co-infecting viruses, resulting in false positives or inaccurate quantification. This highlights the need to fully validate virus-assays prior to use. In contrast to a potential for false positives, ELISA, and other immunoassays, may also be at risk of presenting false negative results. This generally occurs when testing is performed at the early window of infection. At this stage, the quantities of viral antigen may be present at low concentrations, challenging the sensitivity of the ELISA format, potentially leading to erroneous results (Tillmann, 2014; Alexander, 2016). Further to this, ELISA procedures can take several hours to perform. This limits the capacity for rapid testing and results turnaround. However, the multi-well layout of ELISA plates facilitates multiple simultaneous tests, which is ideal for scenarios where high volume testing is required, such as epidemic or pandemic scenarios. In order to improve this turnaround time other antibody-based methods are suggested for virus detection.

#### Blotting Techniques

**FIGURE 7:**
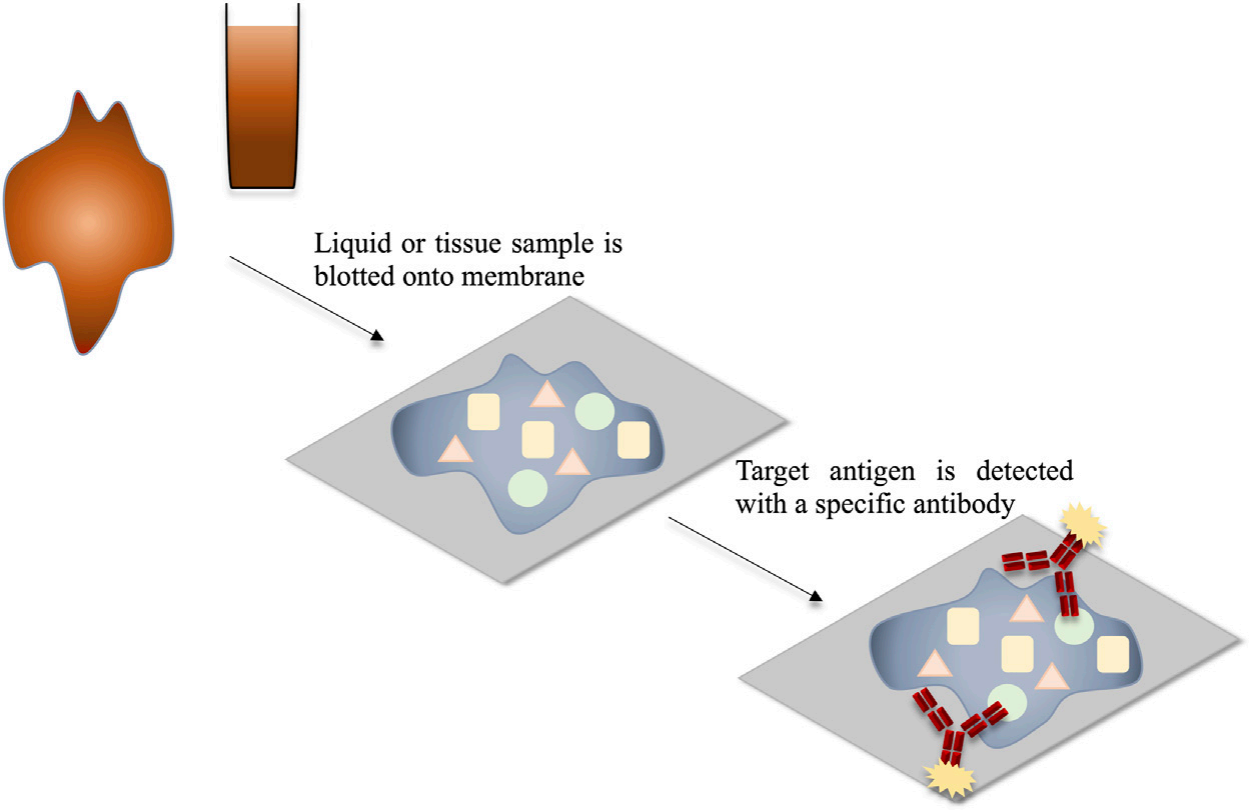
Schematic representation of the detection of viral antigens through dot blot or tissue blot. The mixture of antigens within the sample is blotted onto the membrane and thereby immobilised. The target antigens are detected using target-specific labelled antibodies.

Blotting techniques generally involve antigen detection on the surface of a membrane. The dot blot, or slot blot, is a technique which can be used for the detection of viral antigens from a sample (Li et al., 2010; Gallagher et al., 2011). To do this, usually the suspect sample is blotted onto a membrane, allowed to dry and the membrane is then probed with an anti-virus antibody. This method is cost effective and offers advantages such as the requirement for a small sample volume, the ability to detect virus directly from the sample and the fact that blotted membranes can be stored for a number of days before testing, facilitating the testing of numerous membranes simultaneously. However, the results of dot-blots are mostly qualitative, requiring further instrumentation to perform quantitive analysis which may increase costs. Dot/slot blots can also take several hours to perform (Li et al., 2010; Thiruppathiraja et al., 2011; Fulladolsa Palma et al., 2013). Tissue blots are a variation on the dot/slot blot. These involve pressing of the infected tissue onto the membrane to facilitate virus antigen immobilisation. The dot/slot blot and tissue blot procedures are outlined in Figure 7. Blotting techniques are useful for cheap detection of multiple samples, as many samples can be blotted on the same membrane. However, the time required to produce results is a considerable drawback, as the turnaround may take several hours.

#### Lateral-Flow Immunoassay

A form of lateral flow immunoassay (LFIA), NALFIA, was discussed with respect to its usage in nucleic-acid detection, however, LFIAs are extensively used for detecting virus-associated protein directly from source. While NFLIA function based on the detection of labelled amplicons, standard LFIAs rely on labelled antibodies binding to their cognate antigens. The assays are typically formatted in either sandwich or competitive immunoassay formats (Sajid et al., 2015). The assay result is usually read by way of a colour change at a test line. In sandwich format, a colour change at this line corresponds to a positive result, whereas in competitive format no colour change is indicative of a positive result. Control lines are incorporated into both assays to ensure test validity. The colour change is facilitated by the labelling of antibodies with various molecules. Labels such as gold nanoparticles, coloured latex beads, carbon nanoparticles and magnetic particles facilitate one-step results where a colour change can be observed with the naked eye, facilitating rapid diagnosis. Other labels such as fluorophores, quantum dots and enzymatic labels can also be employed, however these may require additional equipment or steps to demonstrate results which may be qualitative or quantitative (Posthuma-Trumpie et al., 2009; Bahadır and Sezgintürk, 2016; Mak et al., 2016).

For virus detection, LFIAs offer an appealing alternative to methods such as ELISA or blotting methods. One primary advantage is that LFIAs have a turnaround time of minutes, rather than hours. Further to this, performing a LFIA requires minimal sample clean up, having been proven to efficiently detect viral antigens from crude samples, including viral transport media and blood (Ryu et al., 2016; Sastre et al., 2016; Si et al., 2019). Assays can also be performed using saliva samples providing a non-invasive and easy-to-acquire source for viral testing (Yoon et al., 2017). LFIAs are also cost effective, requiring minimal sample volumes to prepare and facilitating diagnosis with the naked eye. However, this also comes with the drawback of only offering qualitative results. To achieve quantitative results, further instrumentation is required (Mak et al., 2016). Another advantage of LFIA is their ease-of-use, which makes the technology accessible to a wide range of users. This can be helpful in scenarios where a high volume of testing is required, as non-specialists can be quickly trained to performs assays.

Overall, immunoassays in viral detection play an essential role. While they may not always offer the same sensitivity as nucleic-acid detection methods, particularly at early stages of infection, they come with the advantage of a reduced cost, reduced complexity and higher utility for use by untrained personnel. These factors are exemplified by the LFIA which can be produced cheaply, has no need for additional equipment and requires little to no training to use. Table 1 provides a comparison between the various nucleic acid detection-based and immunoassay-based methods. The specificity of antibodies in immunoassays is one of the primary challenges. This is exacerbated in virology by the presence of multiple strains of a single virus, or by similar viruses within a genus. Recombinant antibody technology facilitates rapid re-engineering of antibody characteristics such as specificity and offers a means to alter antibodies for reduced cross-reactivity (Ducancel and Muller, 2012; Ma et al., 2019). This could aid in overcoming any specificity-related obstacles. Antibody discovery using recombinant technology also has a faster turnaround when compared to methods such as hybridoma technology (Basu et al., 2019). Therefore, recombinant technology should be considered an essential tool in the development of future viral diagnostics assays for both existing and emergent viral strains.

**TABLE 1:** Summary of the advantages, disadvantages and example applications off nucleic acid-based and immunoassay-based approaches to virus detection.

Method	Advantages	Disadvantages	Examples	Refs.
*qPCR*	Quantitative; highly specific when using well-designed primers; highly sensitive; amenable to high-volume testing	Specialised equipment and trained staff required; relatively long runtime	Dengue; Zika; Chikungunya; Filoviruses; Hantaviruses; Influenza A; Coronavirus	Colombo et al. (2019), Jääskeläinen et al. (2019), Nagy et al. (2019), Nunes et al. (2019), Corman et al. (2020)
*LAMP*	Rapid (30–60 min); sensitive; multiple primer requirements aid in specificity; can be quantitative or qualitative	Requires higher run temperature of 65°C; primer design can be challenging; DNA sample needs denaturation prior to amplification; appears particularly prone to carry-over contamination	Coronavirus; Zika; Dengue; Lassa; Hepatitis B	Jayanath et al. (2018), Lopez-Jimena et al. (2018), Da Silva et al. (2019), Pemba et al. (2019), Huang et al. (2020)
*RPA*	Rapid (20 mins); can operate at room or body temperature; simple primer design; no requirement for DNA denaturation prior to amplification	Low operating temperature can reduce specificity	Respiratory syncytial virus; Zika; Influenza	Wand et al. (2018), Sun et al. (2019), Xi et al. (2019)
*HDA*	Relatively simple primer design; no requirement for DNA denaturation prior to amplification	Cannot operate at ambient temperatures; may be prone to non-specific amplification due to lower operating temperature	Herpes simplex viruses; Varicella-zoster	Teo et al. (2015), Jevšnik et al. (2020)
*RCA*	Multiple formats available (e.g., linear, hyper-branched, multiply primed, padlock probe); at minimum requires only one primer	Product yield can be low depending on amplification method; additional sample manipulation may be required when using padlock-probes in RCA.	Influenza A/B; Human immunodeficiency virus; Ebola; Zika; Dengue; Middle-East respiratory syndrome	Na et al. (2018), Neumann et al. (2018), Ciftci et al. (2020), Soares et al. (2020)
*CRISPR- Cas Nucleic Acid Detection*	Can be used standalone or in conjunction with already established amplification methods; may offer additional specificity as the target must be recognised by both the initial amplification method and guide RNA; relatively rapid (∼60 mins)	Generally requires pre-amplification of target; may require manipulation of sample post-amplification; increasing carry-over contamination risk	Ebola; Epstein-Barr virus; Zika; Dengue; Lassa	Myhrvold et al. (2018), Qin et al. (2019), Barnes et al. (2020), Yuan et al. (2020)
*ELISA*	Multiple formats available (e.g. direct, indirect, competitive, sandwich); assay design made relatively simple by the wide range of commercially available antibodies; amenable to high-volume testing	Long runtime (hours-days); can be affected by sample matrix; different antibodies may exhibit different specificities for target; false negatives may occur in early infection window	Zika; Ebola; Influenza A; Human parechovirus; Hepatitis C; Human immunodeficiency virus	Bystryak and Acharya (2016), Zai et al. (2018), Zhang et al. (2018), Chen et al. (2019), Goto et al. (2020), Patel and Sharma (2020)
*Blotting*	Cheap; can be used with crude samples blotted onto membrane; blots are relatively stable which facilitates multiple sample collection/testing	Only qualitative/semi quantitative; results turnaround of hours-days; limited sensitivity	Respiratory syncytial virus; Dengue; Hepatitis B	Falconar and Romero-Vivas (2013), Gómez et al. (2014), Zhang et al. (2014)
*LFIA*	Rapid (∼10 mins); easy to interpret results; resistant to sample matrix effects; user-friendly	Results generally limited to quantitative/semi-quantitative; does not typically achieve sensitivity comparable to nucleic-acid detection	Zika; Influenza A; Hepatitis B; Dengue; Ebola	Hu et al. (2017), Liang et al. (2017), Wiriyachaiporn et al. (2017), Kumar et al. (2018), Rong et al. (2019)

## Future Directions

While molecular and antibody-based techniques will likely continue to dominate the field of virology, their use should be complemented by engaging with newer technologies, such as next generation sequencing (NGS) platforms. The employment of NGS benefits virology in multiple ways. Firstly, it can act as a stand-alone method for viral diagnostics, if required. NGS is used to sequence genomes, DNAs and RNAs, and can be used to differentiate host genetic information from viral sequences. This, therefore, provides a powerful tool to diagnose viruses, and would be particularly useful in scenarios where patients present with a disease and the causative-pathogen is unidentified (Barzon et al., 2013). While molecular, immuno- and cell-culture methods can be used to identifying unknown pathogens in an investigational “process of elimination” approach, NGS offers a more time-effective method to achieve the same output (Adams et al., 2009). Therefore, while currently established methods may be more rapid in diagnosing known viruses, NGS should be a promising way to reduce time-to-discovery for novel, or unexpected, viral pathogens. Instances of NGS implementation providing a diagnosis to a previously unknown virus, or identifying unexpected viruses in samples, are reported, highlighting its usefulness (Palacios et al., 2008; Prachayangprecha et al., 2014; Naccache et al., 2015; Cordey et al., 2016; Somasekar et al., 2017; Rott et al., 2018) NGS could also be applicable to diagnosing emergent viruses, where no established assays are available for the testing of the virus. Similarly, monitoring of viral levels in a population could also be facilitated by NGS, with particular note to monitoring of emergent or unexpected viral strains. Passive sampling of airborne viruses in crowded areas such as transit networks, schools, day-cares and emergency rooms could be used in conjunction with NGS to assess the viral burden and patterns in particular settings (Choi et al., 2018; Coleman et al., 2018; Prussin et al., 2019; Coleman and Sigler, 2020). In a similar manner, viral vectors or transmitters, such as mosquitoes or bats, can be sampled passively, and routinely tested, to allow surveillance of virus prevalence in the harbouring population (Eiras et al., 2018; Duarte et al., 2020). This could be helpful for predicting disease outbreaks or novel virus emergence, Another benefit of NGS in virology is the promise of expanding viral genome-sequencing data. The paucity of information with regards to viral sequences can make experimental design difficult, for example, designing broad-spectrum amplification primers or identifying conserved antigens can be challenging when not all viral strains are known (Barzon et al., 2011). The capability to sequence many viruses from a population, and hence achieve a more rounded picture of the variation in those viruses, could aid not only in the surveillance of emerging viruses, but in providing additional information to researchers looking to develop novel assays (Rosario and Breitbart, 2011; Radford et al., 2012).

While NGS is a promising technology as a tool for virus discovery, there are challenges to overcome. These include the high costs, the need for both trained staff and bioinformaticians, a potential for poor sensitivity in low viral-load infections and a lack of universal sequence data-analysis tools (Beerenwinkel et al., 2012; Barzon et al., 2013; Massart et al., 2014; Maree et al., 2018). Once addressed, NGS could work in tandem with established techniques to provide a means to identify, characterise and diagnose viral infection.

## Conclusion

Presently, no single method meets every demand of virus detection. Nucleic acid-based detection is highly sensitive and specific. Its drawbacks include the fact that often trained staff are required, associated costs can be high, and carry-over contamination can cause issues in result analysis. In contrast, antibody-based detection is generally cheaper, more robust, and more accessible to untrained users. However, immunoassays do not typically achieve the same sensitivity as nucleic acid detection methods. As such, the decision of whether to employ nucleic acid detection or immunoassays methods will largely be dictated by the requirements of the assay. For example, considerations should include the setting in which the assay will be performed, whether it be laboratory-based or at point-of-care/use, cost of the assay, the urgency of the diagnostic, for example in situations of emergent strains which are causing an epidemic or pandemic, and the sensitivity and specificity requirements of the assay. As NGS becomes more accessible, both nucleic-acid detection and immunoassay methods will benefit from its application. As the use of NGS expands, so too will the number of assays to facilitate the detection of viruses, driven by an increased knowledge of the diversity of viral genomes and their roles.
